# AlPaCas: allele-specific CRISPR gene editing through a protospacer-adjacent-motif (PAM) approach

**DOI:** 10.1093/nar/gkae419

**Published:** 2024-05-25

**Authors:** Serena Rosignoli, Elisa Lustrino, Alessio Conci, Alessandra Fabrizi, Serena Rinaldo, Maria Carmela Latella, Elena Enzo, Gianni Prosseda, Laura De Rosa, Michele De Luca, Alessandro Paiardini

**Affiliations:** Department of Biochemical Sciences “A. Rossi Fanelli”, Sapienza University of Rome, Rome 00185, Italy; Department of Biochemical Sciences “A. Rossi Fanelli”, Sapienza University of Rome, Rome 00185, Italy; Centre for Regenerative Medicine “Stefano Ferrari”, Department of Life Sciences, University of Modena and Reggio Emilia, 41125 Modena, Italy; Centre for Regenerative Medicine “Stefano Ferrari”, Department of Life Sciences, University of Modena and Reggio Emilia, 41125 Modena, Italy; Department of Biochemical Sciences “A. Rossi Fanelli”, Sapienza University of Rome, Rome 00185, Italy; Holostem s.r.l, Via Gottardi 100, 41125 Modena, Italy; Centre for Regenerative Medicine “Stefano Ferrari”, Department of Life Sciences, University of Modena and Reggio Emilia, 41125 Modena, Italy; Department of Biology and Biotechnology Charles Darwin, Sapienza University of Rome, Rome 00185, Italy; Centre for Regenerative Medicine “Stefano Ferrari”, Department of Life Sciences, University of Modena and Reggio Emilia, 41125 Modena, Italy; Centre for Regenerative Medicine “Stefano Ferrari”, Department of Life Sciences, University of Modena and Reggio Emilia, 41125 Modena, Italy; Department of Biochemical Sciences “A. Rossi Fanelli”, Sapienza University of Rome, Rome 00185, Italy

## Abstract

Gene therapy of dominantly inherited genetic diseases requires either the selective disruption of the mutant allele or the editing of the specific mutation. The CRISPR-Cas system holds great potential for the genetic correction of single nucleotide variants (SNVs), including dominant mutations. However, distinguishing between single-nucleotide variations in a pathogenic genomic context remains challenging. The presence of a PAM in the disease-causing allele can guide its precise targeting, preserving the functionality of the wild-type allele. The AlPaCas (Aligning Patients to Cas) webserver is an automated pipeline for sequence-based identification and structural analysis of SNV-derived PAMs that satisfy this demand. When provided with a gene/SNV input, AlPaCas can: (i) identify SNV-derived PAMs; (ii) provide a list of available Cas enzymes recognizing the SNV (s); (iii) propose mutational Cas-engineering to enhance the selectivity towards the SNV-derived PAM. With its ability to identify allele-specific genetic variants that can be targeted using already available or engineered Cas enzymes, AlPaCas is at the forefront of advancements in genome editing. AlPaCas is open to all users without a login requirement and is freely available at https://schubert.bio.uniroma1.it/alpacas.

## Graphical Abstract

**Graphical Abstract:**
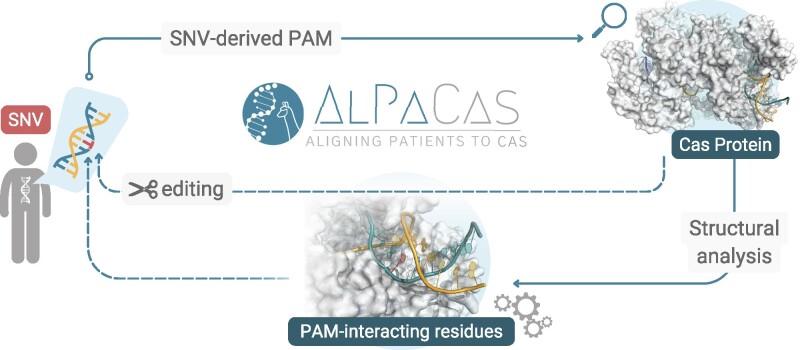

## Introduction

The bacterial-derived defence mechanism known as ‘clustered regularly interspaced short palindromic repeats (CRISPR)/Cas system’, has progressed from early challenges to promising clinical trials for the gene therapy of genetic diseases (1–4). The main components of a CRISPR/Cas system include a Cas nuclease protein and a guide RNA (gRNA). The gRNA binds to a complementary sequence in genomic DNA, adjacent to a ‘protospacer adjacent motif’ (PAM)—a short sequence of 3–8 nucleotides required by Cas to recognize and bind its target DNA properly—and directs the Cas enzyme to a cleavage site, causing DNA strand breaks (5–7). The design of gRNAs complementary to the genomic sequence of interest is one of the strategies utilized to customize the selectivity and specificity of Cas cleavage (8,9). However, the tolerance of mismatches (10,11), leading to unwanted editing of the WT allele (12,13), represents a major hurdle to the clinical translation of CRISPR/Cas-mediated gene editing. PAM–Cas interaction plays a key role in the ATP-independent unwinding of DNA strands for the successive gRNA:DNA heteroduplex formation. Thus, another strategy to increase allele-specificity is based on the recognition of the PAM sequence, to design a gRNA flanked by the PAM that is present only within the allele to be targeted. This emerging strategy proposes to identify a PAM uniquely present in the dominant mutant sequence, enabling precise targeting of the disease-causing allele by a specific Cas (14,15).

The CRISPR-Cas toolbox has been expanded over the years by investigating Cas9/Cpf1 orthologues and other related effector proteins from diverse bacterial species, some of which exhibit different target site specificities (16,17). Moreover, as an alternative approach to modulating the system's selective behaviour, existing Cas9/Cpf1 proteins have been experimentally engineered and refined through rational, structure-based approaches, to enhance their PAM specificities (18–23). Efforts continue to be concentrated on enhancing these enzymes in order to enable versatile genome engineering, thereby granting access to every sequence in the genome. This emphasis is particularly crucial because many pathogenic alleles lack mutations that create PAM sites recognizable by the current Cas repertoire, rendering them ‘undruggable’. Consequently, it becomes pivotal to provide tools for the effective screening and analysis of the continuously expanding repertoire of Cas enzymes.

In this context, we developed AlPaCas, ‘Aligning Patients to Cas’. AlPaCas is a computational pipeline designed for sequence-based identification and structural analysis of SNV-derived PAMs. In this context, a ‘SNV-derived PAM’ is the occurrence where an SNV aligns with a PAM pattern, discriminating the mutant allele with respect to the wild-type. When provided with an input such as gene/SNV data (HGNC GeneSymbol, NCBI Gene-ID, VCV-identifiers or raw sequences), AlPaCas can detect the presence of SNV-derived PAMs. Optionally, it conducts a structural analysis of the Cas/PAM interaction, offering insights for potential optimization. To date, AlPaCas is the only available tool accomplishing such tasks, given that accessible platforms, i.e. web servers, for allele-specific CAS system engineering only aim at gRNA design (24).

Finally, AlPaCas features a user-friendly interface, making it accessible to users with different levels of expertise and obviating the necessity for manual analysis. With the ability to eliminate barriers in manual data processing, AlPaCas represents a valuable resource in the field of precision gene editing for both basic research and clinical application.

## Material and methods

### Web-server implementation

AlPaCas is deployed on Apache 2, as a freely available and user-friendly web server at https://schubert.bio.uniroma1.it/alpacas/. The frontend is built using pure HTML (v. 5), CSS (v. 3) and JavaScript (ECMAScript 2020). The server's environment leverages the Flask framework (version 3.0) to handle backend processes. The backend computational processes are implemented in Python 3. AlPaCas is hosted on a dedicated Linux machine of high performance (GPU RTX3070—12th Gen Intel(R) Core(TM) i9-12900KF), ensuring reliable performance and availability.

### Protocols

#### Local databases management

AlPaCas relies on the integration of information from the publicly available databases ClinVar (25), for SNV-annotations, and NCBI Genome (26), for genomic sequences. A local version of each database has been adapted for convenient accessibility through Structured Query Language (SQL) queries. We developed methods to manage the conversion of databases from their distributed, tab-separated format into SQL databases, catering to handling large volumes of data. This management approach significantly speeds up the analysis and reduces the likelihood of server-side call interruptions. However, to keep the databases up to date, the entire procedure has been automated with in-house Python scripts, which are processed on a monthly basis through the Flask scheduling module. For convenience, also the structural database of Cas enzymes has been adapted as a SQL-accessible database. The Python module sqlite3 is used to access the required information during the processing steps.

#### Search protocol

The protocol operates by cross-referencing a source of SNVs with a compendium of established PAM patterns. For every SNV, the protocol scans the sequences—both wild-type and mutated, as well as their forward and reverse orientations—flanking the site of mutation, utilizing the list of PAM patterns. The sequence considered for screening includes the mutation itself and it extends upstream and downstream for a length equal to the PAM pattern analysed. Therefore, the nucleotides flanking the mutation also take a role in the selective targeting of the alleles. As an example, with NG-Cas9 (PAM pattern: NG) and a mutation N→G in the SNV, if the downstream flanking nucleotide is also a G, NG-Cas9 could position the PAM ‘N’ nucleotide at the mutation site, and the ‘G’ at the adjacent position, thus not providing selectivity between the wild-type and mutant alleles. For the pattern-matching operation, the system employs regex-based string-matching algorithms. During this phase, the system maintains a dynamic hash table data structure to store and quickly retrieve the occurrence of each PAM pattern within the mutant and wild-type sequences. The goal is to identify PAM patterns that exclusively match the mutant sequences (both forward and reverse), while ensuring no matches with the wild-type counterparts.

#### Structural analysis protocol

The structural manipulation of Cas proteins is expedited through the incorporation of the BioPython module (27), which facilitates the identification of PAM-interacting residues by adhering to user-specified threshold parameters. Upon detecting each PAM-interacting residue, a tailored set of potential amino acids is generated, marked as prospective mutation candidates. This selection is curated based on the scores obtained from the BLOSUM62 substitution matrix. The ‘ProteinDesign’ functionality within UniDesign (28) is used to mutate the residues and predict the ΔΔG values for each mutated residue. To comprehensively score all the proposed mutants, given that UniDesign only provides the top-scored one, the system employs an iterative approach, systematically removing the top-scored mutant in each cycle to allow the evaluation of subsequent candidates. UniDesign was selected for scoring purposes due to its recent benchmarking success in evaluating Cas-PAM interactions, particularly benefiting from the integration of newly implemented nucleotide topologies derived from the all-atom CHARMM36 force field (29). Cas enzyme kinetics (30–32), suggest the possibility of interpreting a decrease in ΔΔG in the Cas-PAM interaction, as a clue for increased enzyme activity. This interpretation aligns with the canonical notions of enzyme's Km and ΔΔG correlation (33).

#### Evolutionary conservation of PAM-interacting residues

The sequence of the Cas protein under study is used as a query sequence against the UniProt Reference Clusters (UniRef) database (34), to look for homologs within a certain threshold of sequence identity (30–99 by default). The retrieved homologous sequences are aligned with MUSCLE (35) and subjected to conservation analysis using the CAMPO algorithm (36). This algorithm provides a normalized metric for residue conservation, offering a conservation index ranging from 0, low conservation, to 1, high conservation.

## Results

### Web-server description

**Figure 1.**
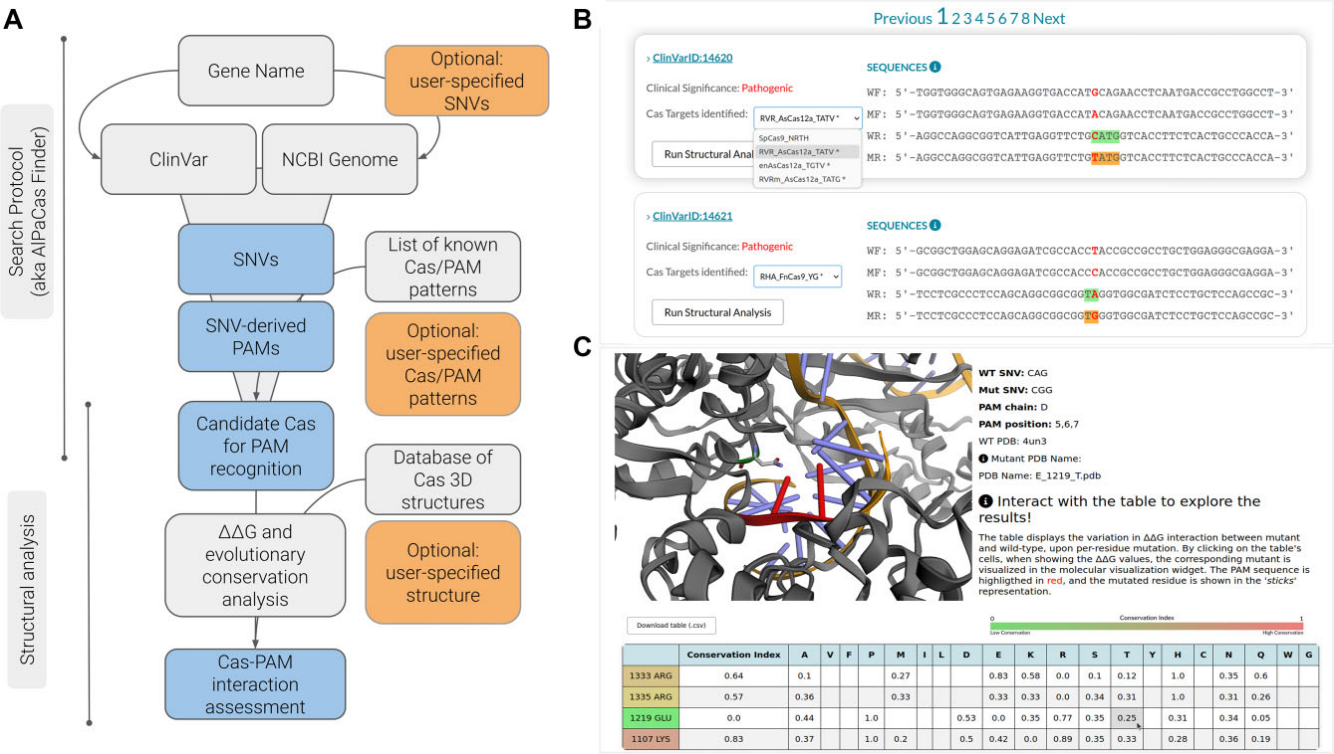
AlPaCas workflow and appearance. (**A**) The figure shows the sequential stages of the AlPaCas workflow, which is conceptualized in two main phases: the search protocol, represented by AlPaCas Finder, and the subsequent structural analysis. Each phase can be further elucidated with substeps, where input/methods of the analysis are depicted in grey, while optional inputs are accentuated in orange. The outputs of each step are visually presented in blue. The results of each step have dedicated results pages, such as an intuitive interface to scroll across the identified SNV-derived PAMs (**B**) and an interactive interface to explore the results of the structural analysis (**C**).

AlPaCas consists of two consecutive protocols (Figure 1A): (i) the search protocol (aka AlPaCas Finder), which aims to identify SNV-derived PAMs by cross-referencing SNV sources with an available list of PAM sequences associated with respective Cas proteins (Table 1; Figure 1B); (ii) the structural analysis protocol, designed to explore structural basis of SNV-derived PAM-dependent target DNA recognition (Figure 1C).

**Table 1.** Summary of the Cas proteins represented in AlPaCas's databases. A total of 39 Cas proteins, 22 natural and 17 variants, are represented. Among which we have 39 3D-structures distributed as follows: (SpCas9: 3; SpCas9-VQR: 1; SpCas9-VRER: 2; SpCas9-EQR: 2; SpCas9-NG: 1; xCas9: 6; Nme1Cas9: 2; Nme2Cas9: 2; FnCas9: 2; FnCas9-RHA: 1; SaCas9-KKH: 2; CjCas9: 2; AceCas9: 2; AsCas12a: 1; AsCas12a-RVR: 1; AsCas12a-RR: 1; LbCas12a: 4; Cas14: 2; Cas12c1: 1; Cas12c2: 1)

Name	Organism	Uniprot-ID/variant	Subtype	PAM pattern	Structure
SpCas9	*S. pyogenes*	Q99ZW2	II-A	NGG^1^	4UN3 (40), 5Y36 (41), 5FQ5 (42)
SpCas9	*S. pyogenes*	VQR	II-A	NGA	5B2R (43)
SpCas9	*S. pyogenes*	VRER	II-A	NGCG	5B2T (43), 5FW3 (44)
SpCas9	*S. pyogenes*	EQR	II-A	NGAG	5B2S (43), 5FW2 (44)
SpCas9	*S. pyogenes*	NG	II-A	NG	6AI6 (45)
SpCas9	*S. pyogenes*	NRRH	II-A	NRRH	−
SpCas9	*S. pyogenes*	NRCH	II-A	NRCH	−
SpCas9	*S. pyogenes*	NRTH	II-A	NRTH	−
xCas9	*S. pyogenes*	−	II-A	NGN	6K4P, 6K4Q, 6K4S, 6K4U (46), 6AEB, 6AEG (47)
SaCas9	*S. aureus*	J7RUA5	II-A	NNGRRT	5AXW, 5CZZ (48)
SaCas9	*S. aureus*	KKH	II-A	NNNRRT	−
St1Cas9	*S. thermophilus*	Q03JI6	II-A	NNRGAAW^2^	−
St3Cas9	*S. thermophilus*	G3ECR1	II-A	NGGNG	−
SpasCas9	*S. pasteurianus*	F5 × 275	II-A	NNGTGA	−
TdCas9	*T. denticola*	Q73QW6	II-A	NAAAAC	−
Nme1Cas9	*N. meningitidis*	C9 × 1G5	II-C	NNNNGATT	6JDV, 6KC8 (49)
Nme2Cas9	*N. meningitidis*	A1IQ68	II-C	NHDTCCA	6JFU, 6JE3 (49)
CjCas9	*C. jejuni*	Q0P897	II-C	NNNVRYM	5X2H, 5X2G (50)
AceCas9	*A. cellulolyticus*	A0LWB3	II-C	NNNCC	6WBR,6WC0 (51)
FnCas9	*F. novicida*	A0Q5Y3	II-B	NGG^3^	5B2O, 5B2P (51)
FnCas9	*F. novicida*	RHA	II-B	YG	5B2Q (52)
FnCas12a (Cpf1)	*F. novicida*	A0Q7Q2	V-A	TTV	−
AsCas12a	*Acidaminococcus*sp.	U2UMQ6	V-A	TTTN^4^	5B43 (53)
AsCas12a	*Acidaminococcus*sp.	RVR	V-A	TATV	5XH6 (54)
AsCas12a	*Acidaminococcus*sp.	RR	V-A	TYCV	5XH7 (54)
enAsCas12a	*Acidaminococcus*sp.	TGTV	V-A	TGTV	−
enAsCas12a	*Acidaminococcus*sp.	VTTV	V-A	VTTV	−
enAsCas12a	*Acidaminococcus*sp.	TTTT	V-A	TTTT	−
enAsCas12a	*Acidaminococcus*sp.	TTCN	V-A	TTCN	−
AsCas12a	*Acidaminococcus*sp.	RVRm	V-A	TATG	
LbCas12a	*L. bacterium*	A0A5S8WF58	V-A	TTTN^5^	5XUS, 5XUT, 5XUU, 5XUZ (55)
MbCas12a	*M. bovoculi*	A0A3F3CDD6	V	TTTN	−
AacCas12b (c2c1)	*A. acidoterrestris*	T0D7A2	V-B	DTTD	−
BthCas12b	Brevibacillus sp.	A0A9 × 7XSH8	V-B	ATTN	−
DpbCas12e	*Deltaproteobacteria*	A0A357BT59	V-E	TTCN	−
Cas12e	*Planctomycetes*	A0A1G3BXR9	V-E	TTCN	−
Cas14a1 (Cas12f)	*Uncultured archaeon*	A0A482D308	V-F	TTTR^6^	7L49 (56), 7C7L (56)
Cas12c1	*P. muris*	A0A9 × 9ZA50	V-C	TG	7VYX (57)
Cas12c2	*P. muris*	*NA*	V-C	TN	7V94 (58)

Less-preferred PAMs: ^1^ NAG, NGA; ^2^ NNGGAAW, NNGATAG, NNTCTTA, NNCAATA; ^3^ NAG, NGA; ^4^ VTTV, YCCA, TCTV, TTCV, GTTV, GCTV; ^5^ TCTA, TCCA, CCCA; ^6^ YTCA.

AlPaCas is designed to ensure a user-friendly approach, with minimal input requirements. When using AlPaCas, the user has two primary input options to start the analysis: (i) GeneSymbol (HGNC nomenclature) or GeneID, if interested in analysing all ClinVar-annotated SNVs for a specific gene; (ii) VariationID (VCV identifier), if interested in focusing on a single, or a few, specific variants annotated in ClinVar. AlPaCas also has the functionality to include user-provided SNVs, thereby covering SNVs that are not annotated in ClinVar. The use of local databases for PAM patterns and Cas protein structures streamlines the analysis by eliminating the need for additional information, ensuring a straightforward process. Nonetheless, for enhanced flexibility, users are given the option to incorporate unlisted Cas proteins into the system, with the choice of including them, with or without an associated structure.

After running AlPaCas Finder, the user can delve into the results via the dedicated results page (Figure 1B). Each panel on this page provides detailed information on each variation associated with the user's input query. The panels display information for each identified SNV as follows: (i) a direct link to the corresponding ClinVar page; (ii) the ‘Clinical Significance’ as reported in ClinVar; (iii) a widget showing the Cas proteins found to target the specific SNV-derived PAM. Some hints are present in widget (iii) to guide the users towards an optimal selection. Cas enzymes that can discriminate between the SNV-derived PAM and wild-type with a specific nucleotide, according to their annotated PAM pattern, are identified with a symbol ‘*’ next to their name. For instance, a C→G mutation could potentially be discerned by an ‘*R’* pattern, but it is preferable to utilize a Cas nuclease specifically recognizing a G in that position. Moreover, Cas enzymes are ordered according to a ‘selectivity score’ assigned to each PAM pattern, which is weighted based on the number of specific nucleotides recognized (*N* = 0.25; V, H, D, B = 0.33; M, R, W, S, Y, K = 0.50; A, C, G, T = 1.00) and the length of the PAM pattern (17,18). The widget allows for a dynamical visualization, illustrating how a chosen Cas could potentially interact with the sequence. The area around the mutation targeted by the chosen Cas and the corresponding WT reference sequence are clearly differentiated, to provide a clear contrast between the targeted region and the unaltered sequence.

Optionally, when the 3D-structure of a Cas enzyme potentially acting upon the SNV-derived PAM is available, the user can access the structural analysis module, selecting one of the available Cas proteins and setting the threshold (Å) for the identification of PAM-interacting residues within the binding site. The latter residues are analysed for their evolutionary conservation, which may suggest residues with catalytic functions, hence not suited for engineering proposals. Pre-computed evolutionary conservation indices for PAM-interacting residues in each structure are available, offering users the choice to streamline the analysis process. Alternatively, a threshold of percentage of sequence identity must be provided for scoring the evolutionary conservation. Following the evolutionary conservation analysis, the Unidesign algorithm is used to mutate the identified Cas residues and quantify the PAM interaction energy (ΔΔG) difference between the original residues and the suggested amino acid replacements. The choice of Unidesign is based on its validated performance in accurately modelling Cas–PAM interactions (28).

The outcome of the structural analysis, comprising the list of suggested amino acid mutations to enhance the selectivity of Cas enzymes for the SNV-derived PAM, can be scrutinized on the separated interactive page. The latter features a graphical viewer, along with a plot and a table for the detailed visualisation of the ΔΔG values for each mutated residue (Figure 1C). Negative ΔΔG values suggest a favourable interaction with the PAM. By clicking on the ΔΔG value shown in the table, the corresponding mutant is visualised in the molecular visualisation widget, allowing the user to analyse the 3D-structure of the mutant and provide a rationale for the increased affinity. The evolutionary conservation index is also shown for each PAM-interacting residue, both on the plot, and on the interactive table. Noteworthy, it is also possible to download the entire set of Protein Data Bank (PDB) mutants created in this step.

### Cas systems and SNVs in AlPaCas

In implementing AlPaCas, we adopted the currently used classification of Cas enzymes (37,38): class 1, types I, III, IV and class 2, types II, V and VI. Since class 1 proteins are not used in gene editing approaches—they show a fairly complex quaternary structure (39)—we included only class 2 Cas enzymes (both natural, and engineered or variant), which possess a single subunit (Table 1). Among the plethora of class 2 Cas orthologs, type-II-CRISPR-Cas9 (from now on CRISPR-Cas9) and type-V-CRISPR-Cpf1 (Cpf1, also known as Cas12a) emerged as the most efficient and easy-to-handle systems for synthetic genome editing (18,59–62). From the literature (17), 39 Cas proteins of class 2 (type II and V) with annotated PAM patterns have been identified, and inserted in the AlPaCas database.

**Figure 2.**
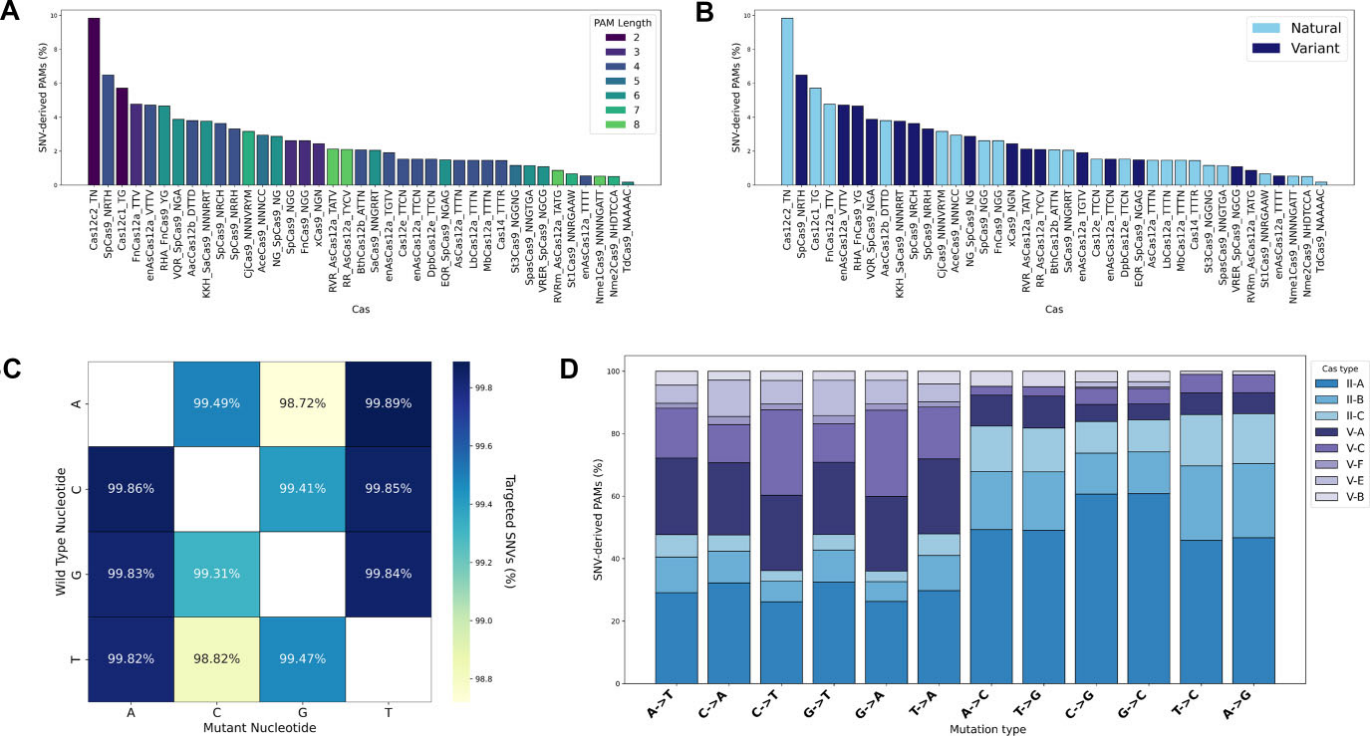
Analysis of 108614 ‘Pathogenic’ or ‘Likely pathogenic’ SNVs annotated in Clinvar (February 2024). In this context, a SNV refers to any variation analysed, while a SNV-derived PAM denotes the matching event between a SNV and a PAM pattern, specifically when it discriminates for the mutant allele. Bar plots (Y axis) showing the percentage of SNV-derived PAMs (number of SNV-derived PAMs for each Cas over the total number of SNV-derived PAMs) recognized by each Cas type (X axis) are colour-mapped by PAM length (**A**) or Cas source, i.e. natural or engineered (**B**). The heatmap in (**C**) shows the percentage of the 108614 SNVs which are targetable, broken down by mutation type. Each cell annotates the percentage of targetability for a specific mutation, from a wild type nucleotide to a mutant nucleotide, as indicated by the rows and columns, respectively. (**D**) A comparative visualization of the cumulative percentages of SNV-derived PAMs targeted by each Cas type is presented for each mutation type.

To assess the potential coverage of the obtained database, we then screened 108 614 SNVs of 4882 genes, namely those annotated as ‘Pathogenic’ or ‘Likely pathogenic’ in ClinVar (February 2024). A high percentage of SNVs (98.5%) generates at least one SNV-derived PAM, showcasing the potential of the approach. Among the identified SNV-derived PAMs, 47.1% are recognized by natural Cas, while 52.9% by engineered Cas, with a prominence for Cas12c2 (Figure 2A), likely for its short PAM sequence (Figure 2B). The less targetable SNVs, i.e. SNVs for which no SNV-derived PAMs have been found, feature G/C mutations (Figure 2C). Figure 2D shows how the identified SNV-derived PAMs are covered by different Cas types, per type of mutation. The data reported here are intended to comprehensively showcase the extent to which the diverse PAM sequences employed cover the spectrum of SNV-derived PAMs identified.

**Figure 3.**
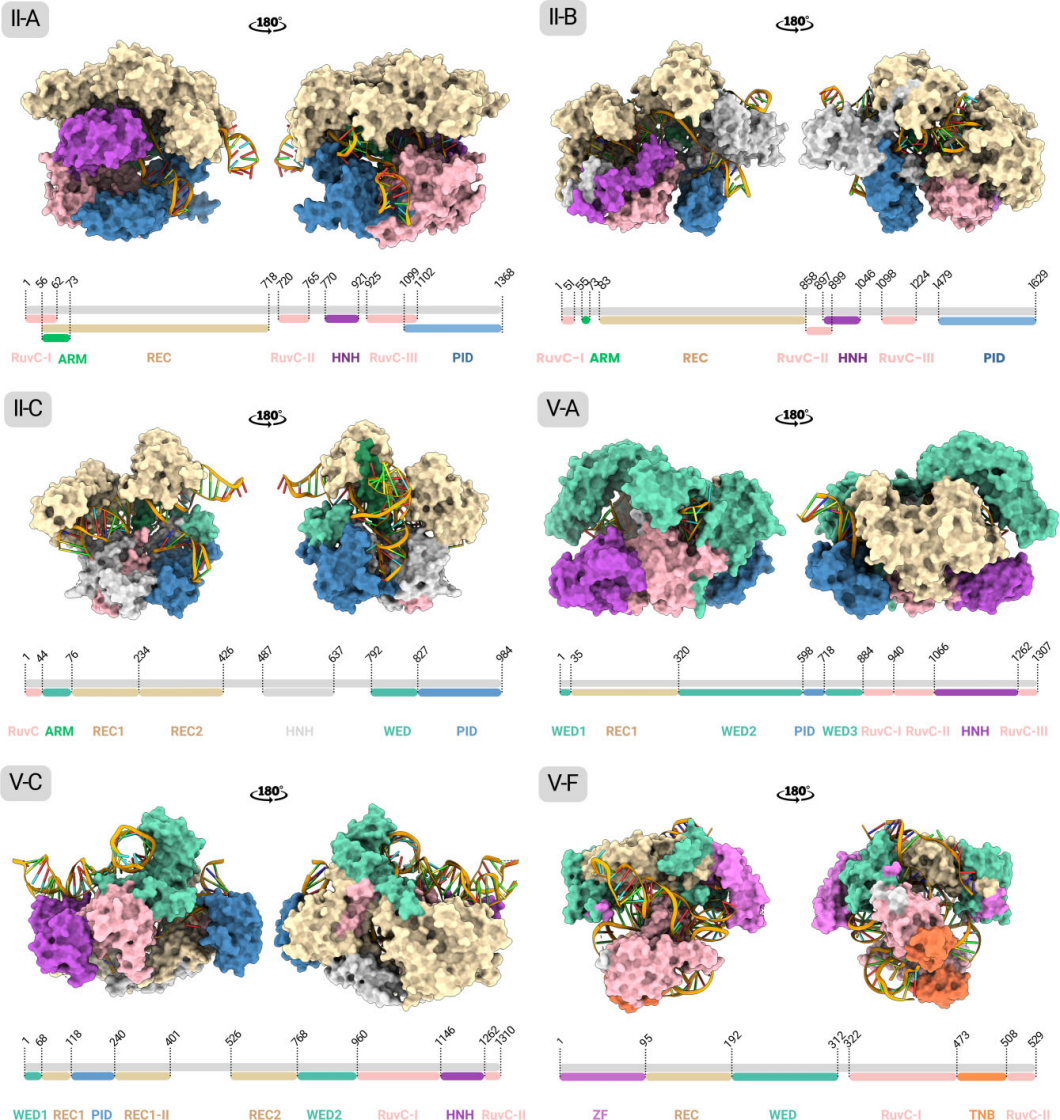
Domain-mapped Cas proteins of the AlPaCas structural database. The image shows the selected conformations of each Cas type, to be submitted to the structural analysis protocol. A representative structure has been chosen for each subtype as follows; II-A: 4UN3, Q99ZW2 (44); II-B: 5B2Q, A0Q5Y3 (56); II-C: 5 × 2H, Q0P897 (54); V-A: 5B43, U2UMQ6 (57); V-C: 7VYX, A0A9 × 9ZA50 (61); V-F: 7C7L, A0A482D308 (60).

Following the search protocol, depending on the availability of the 3D structure (Table 1), the Cas residues interacting with the SNV-derived PAM are analysed in terms of evolutionary conservation and ΔΔG. Starting from the obtained Cas database, an exhaustive search in PDB (63) has been carried out, to only select experimentally-derived structures in complex with the corresponding PAM sequence. After applying this filtering process, we identified 39 suitable 3D-structures (Table 1), covering the type II, subtypes A, B, C and type V, subtypes A, C, F (Figure 3). For every 3D-structure, the conservation scores were calculated for each residue within the multiple sequence alignment (MSA) of Cas protein homologs, which were obtained from the UniRef database (34), across a sequence identity range of 30–99%. The conservation scores can be optionally taken into consideration as indicative of functional residues whose modification could potentially disrupt the Cas catalytic activity. It is advisable that the suggested mutations should be thoughtfully considered as a mere indication to guide the engineering of Cas proteins specifically customized for PAM interaction, and that the prediction of Cas mutation residues based on the algorithm provided in this study should be considered a supplementary and not mandatory step.

### Case study

**Figure 4.**
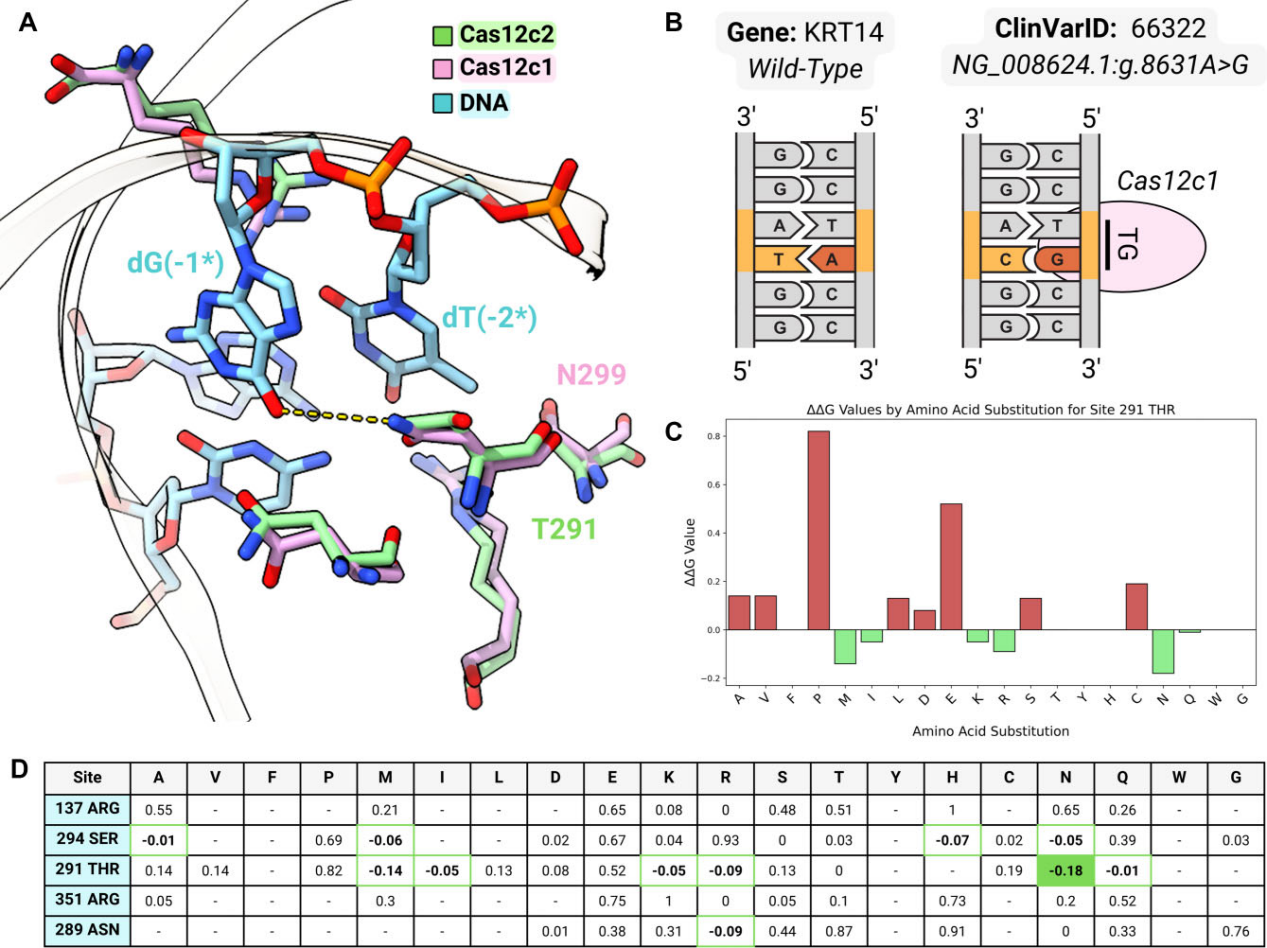
Case study. (**A**) To rationalize the distinct PAM recognition pattern of two close homologs, Cas12c1 and Cas12c2, the 3-D structures, respectively 7vyx (pink) and 7v94 (green), in complex with the DNA (skyblue) have been superimposed and visualised with ChimeraX. Asn299 of Cas12c1, which forms an hydrogen bond (dashed yellow line) with O6 of dG(-1*) overlaps with Thr291 of Cas12c2. (**B**) A SNV associated with EBS type 1A (ClinVar-ID: 66322; Gene: KRT14) analysed with AlPaCas shows the formation of a SNV-derived PAMs ‘TG’, targetable by Cas12c1. The sequences flanking the nucleotide of interest, which is highlighted in red, are illustrated for both the wild-type and the mutant. The PAM recognition pattern ‘TG’ is mapped in correspondence with the target sequence. (**C**, **D**) The ΔΔ*G* values, computed with the structural analysis protocol, on Cas12c2 are reported for Thr291 (C) and for all PAM-interacting residues (D).

Epidermolysis bullosa simplex (EBS), the most common form of epidermolysis bullosa, is a group of rare genetic skin diseases characterised by intraepidermal blisters (64). Approximately 75% of EBS patients carry dominant mutations in *KRT5* and *KRT14—*the genes encoding keratin 5 (K5) and keratin 14 (K14), respectively—that lead to disruption of the intermediate filaments of basal keratinocytes (65,66). The most severe EBS subtype, formerly identified as Dowling-Meara type 1A (EBS1A OMIM: 131760, (67)), is characterized by generalized blistering occurring upon minimal mechanical pressure, typically beginning at birth. EBS1A exhibits a distinctive blistering pattern resembling a herpetiform arrangement, accompanied by lesions featuring crusting and necrosis, often with inflammatory plaques. The CRISPR-Cas9 system holds great promise to correct this skin disorder by initiating a non-homologous end-joining (NHEJ) repairing process specifically on the mutant allele while keeping the wild-type allele intact. We analysed pathogenic SNVs annotated to the KRT14 gene, focusing on those reported to be related to EBS1A, which account for a total of 10 SNVs (ClinVar-IDs: 14611, 14612, 14613, 14619, 14622, 14628, 66322, 66339, 1048026, 1048024). In 7 cases, we were able to identify a SNV-derived PAM that is targeted by Cas12c1 or Cas12c2 enzymes (Supplementary Table S1). Cas12c1 and Cas12c2, featuring a single RuvC nuclease domain, are able to recognize minimal PAM sequences (58). The available 3D-structures (57,58) show that their different PAM specificity, i.e. ‘5′-TG-3′’ for Cas12c1 versus ‘5′-TN-3′’ for Cas12c2 (where ‘N’ stands for any nucleotide), can be rationalized by the presence of a key amino acid replacement, i.e. T291N (57, Figure 4A), at the PAM binding site.

Thr291 is indeed in contact with the dG(-1*) of the PAM sequence. The T291N mutation enhances specificity towards the ‘TG’ PAM compared to ‘TN’, since Asparagine is able to form an additional hydrogen-bond with O6 of dG(-1*), greatly increasing the selectivity toward this nucleotide, as also discussed in literature (57).

Therefore, to validate the ability of AlPaCas to suggest this amino acids replacement, to enhance the selectivity of Cas12c2 from 5′-TN-3′ to 5′-TG-3′, we focused on ClinVar-ID: 66322, featuring an A→G mutation (‘NG_008624.1:g.8631A > G’, Figure 4b) in a wild-type pattern ‘TA’. Using the structural analysis protocol of AlPaCas with ClinVarID 66322, and Cas12c2 as the initial 3D-structure, we identified five residues in contact (threshold 4.0 Å) with the ‘TG’ SNV-derived PAM, namely R137, S294, T291, R351, N289. Among the suggested mutations, the most promising in terms of ΔΔ*G* value was T291N (Figure 4C, D), which corresponds to the homologous Asparagine residue (i.e. Asn299) of Cas12c1 (Figure 4A).

### Interpretation of the results and limitations of the approach

Incorporating alternative PAM patterns for the same Cas into the AlPaCas database acknowledges the inherent Cas promiscuity documented in the literature, as well as the variability in some Cas-PAM interactions (68,69), thus enhancing the tool's versatility. However, this expansion comes with a caveat of potentially ‘over-representing’ targetable PAMs, particularly considering that certain patterns can significantly vary in their affinity for a given Cas. Conversely, representing a specific PAM pattern to reflect a well-defined Cas nuclease's preference may impose overly stringent criteria and result in the loss of important information. Therefore, to optimize the potential of AlPaCas, users should carefully consider how the classical representation of PAM patterns may impact the analysis and alter the interpretation of the results. For example, given the inherent stringency of the PAM pattern, users are encouraged to explore alternative, less frequent PAM preferences to ensure comprehensive coverage.

Another crucial aspect to consider when critically analysing AlPaCas results is PAM selectivity. It is essential to ascertain whether the SNV-derived PAM exhibits specific differentiation towards a particular nucleotide mutation (17,18). This discrimination profoundly affects the efficacy and specificity of the Cas protein in targeting the desired SNV. In this regard, the selectivity score associated with a given PAM pattern could help users to make informed decisions when selecting a Cas protein for targeting specific SNVs. A higher selectivity score indicates greater specificity, enhancing the likelihood of successful SNV targeting.

Sequence logos offer an alternative, visually intuitive way compared to patterns, to illustrate PAM sequence preferences of Cas nucleases, making them well-suited for showcasing Cas selectivity. However, their current implementation poses challenges due to the limited availability of experimental data. While sequence logos are not currently integrated into AlPaCas due to this data scarcity, it's promising to note that they will be included once more experimental results become accessible. This forward-looking approach demonstrates AlPaCas's commitment to incorporating the most effective tools for analysing Cas selectivity as the field progresses.

## Conclusions

Recently, the proof of concept that a SNV-derived PAM could overcome the poor allele selectivity of gRNA in gene editing has been exploited in clinical studies and trials for treating genetic diseases. However, the development of computational tools assisting in the identification of SNV-derived PAMs and the corresponding Cas enzymes, is still an unmet need.

By featuring a user-friendly web server that facilitates the automated screening and analysis of SNV-derived PAMs, AlPaCas addresses this problem, expanding the range of targetable PAM sequences. Due to the paucity of experimental data that is available in this context, it is still hard to benchmark the rational engineering of Cas proteins on a large scale towards the recognition of any desired PAM. However, the promising preliminary results obtained through the implementation of state-of-the-art tools for the evolutionary conservation and ΔΔ*G* of Cas/PAM interaction, along with the ever-increasing availability of novel characterized Cas enzymes and experimental studies reporting on their successful application in gene editing, pave the way for the straight translation of Cas-based novel therapeutics into personalized medicine.

## Supplementary Material

gkae419_Supplemental_File

## Data Availability

AlPaCas is open to all users without a login requirement and is freely available at https://schubert.bio.uniroma1.it/alpacas.
