# Correction to ‘AlPaCas: allele-specific CRISPR gene editing through a protospacer-adjacent-motif (PAM) approach’

**DOI:** 10.1093/nar/gkae1285

**Published:** 2024-12-19

This is a correction to: Serena Rosignoli, Elisa Lustrino, Alessio Conci, Alessandra Fabrizi, Serena Rinaldo, Maria Carmela Latella, Elena Enzo, Gianni Prosseda, Laura De Rosa, Michele De Luca, Alessandro Paiardini, AlPaCas: allele-specific CRISPR gene editing through a protospacer-adjacent-motif (PAM) approach, *Nucleic Acids Research*, Volume 52, Issue W1, 5 July 2024, Pages W29–W38, https://doi.org/10.1093/nar/gkae419

Acknowledgement of funding support from Fondazione Telethon [grant number GGP20088] was omitted from the originally published version and has been added.

