# Supplementary material for: AlPaCas: allele-specific CRISPR gene editing through a protospacer-adjacent-motif (PAM) approach
Source: Nucleic Acids Res. 2024 May 25;52(W1):W29–38. doi: 10.1093/nar/gkae419 (PMC11223865; doi:10.1093/nar/gkae419)
Supplement: gkae419_Supplemental_File [file gkae419_supplemental_file.pdf]

AlPaCas: Allele-specific CRISPR Gene Editing through a Protospacer-Adjacent-Motif (PAM) approach

Serena Rosignoli[1], Elisa Lustrino[1], Alessio Conci[2], Alessandra Fabrizi[2], Serena Rinaldo[1], Maria Carmela Latella[3], Gianni Prosseda[4], Laura De Rosa[2], Michele De Luca[2,*†], Alessandro Paiardini[1,*†].

Supplementary Table 1. Case study: summary of the AlPaCas Finder analysis on *KRT14* gene. In the table are reported the SNV-derived PAMs, and respective targeting Cas, identified for the 10 SNVs (VariationID) associated with EBS1A. Those targetable by Cas12c1 or Cas12c2, are highlighted.

VariationID	Name	Strand	Wild Type	Cas	PAM Pattern	SNV-derived PAM
14611	NM_000526.5(KRT14):c.1151T>C (p.Leu384Pro)	Rv	5’-C**A**G-3’	SpCas9	NGG	5’-C**G**G-3’
		Rv	5’-C**A**G-3’	FnCas9	NGG	5’-C**G**G-3’
		Rv	5’-C**A**-3’	RHA_FnCas9	YG	5’-C**G**-3’
14612	NM_000526.5(KRT14):c.373C>T (p.Arg125Cys)	Rv	5’-C**G**G-3’	SpCas9	NAG	5’-C**A**G-3’
		Rv	5’-GGC**G**-3’	SpCas9	NRCH	5’-GGC**A**-3’
		Rv	5’-C**G**G-3’	FnCas9	NAG	5’-C**A**G-3’
		Fw	5’-**C**G-3’	Cas12c1	TG	5’-**T**G-3’
		Fw	5’-**C**G-3’	Cas12c2	TN	5’-**T**G-3’
14613	NM_000526.5(KRT14):c.374G>A (p.Arg125His)	Rv	5’-**C**G-3’	Cas12c1	TG	5’-**T**G-3’
		Rv	5’-**C**G-3’	Cas12c2	TN	5’-**T**G-3’
14619	NM_000526.5(KRT14):c.356T>C (p.Met119Thr)	Rv	5’-TGC**A**-3’	VRER_SpCas9	NGCG	5’-TGC**G**-3’
14622	NM_000526.5(KRT14):c.1256T>A (p.Leu419Gln)	Rv	5’-**A**G-3’	RHA_FnCas9	YG	5’-**T**G-3’
		Rv	5’-**A**G-3’	Cas12c1	TG	5’-**T**G-3’
		Rv	5’-**A**G-3’	Cas12c2	TN	5’-**T**G-3’
14628	NM_000526.5(KRT14):c.368A>G (p.Asn123Ser)	Fw	5’-CA**A**-3’	SpCas9	NAG	5’-CA**G**-3’
		Rv	5’-CA**T**T-3’	SpCas9	NRCH	5’-CA**C**T-3’
		Fw	5’-CA**A**-3’	FnCas9	NAG	5’-CA**G**-3’
		Fw	5’-CA**A**TGA-3’	SpasCas9	NNGTGA	5’-CA**G**TGA-3’
66322	NM_000526.5(KRT14):c.1244A>G (p.Tyr415Cys)	Rv	5’-GG**T**A-3’	SpCas9	NRCH	5’-GG**C**A-3’
		Fw	5’-T**A**-3’	Cas12c1	TG	5’-T**G**-3’
66339	NM_000526.5(KRT14):c.346A>T (p.Lys116Ter)	Fw	5’-AG**A**A-3’	SpCas9	NRTH	5’-AG**T**A-3’
		Rv	5’-T**T**CT-3’	SpCas9	NRCH	5’-T**A**CT-3’
		Fw	5’-GTGAG**A**-3’	SaCas9	NNGRRT	5’-GTGAG**T**-3’
		Fw	5’-**A**A-3’	Cas12c2	TN	5’-**T**A-3’
1048026	NM_000526.5(KRT14):c.1223T>A (p.Leu408Gln)	Rv	5’-**A**G-3’	RHA_FnCas9	YG	5’-**T**G-3’
		Rv	5’-**A**G-3’	Cas12c1	TG	5’-**T**G-3’
		Rv	5’-**A**G-3’	Cas12c2	TN	5’-**T**G-3’
1048024	NM_000526.5(KRT14):c.1144G>T (p.Glu382Ter)	Fw	5’-AG**G**A-3’	SpCas9	NRTH	5’-AG**T**A-3’
		Rv	5’-T**C**CT-3’	SpCas9	NRCH	5’-T**A**CT-3’
		Fw	5’-TGGAG**G**-3’	SaCas9	NNGRRT	5’-TGGAG**T**-3’
		Fw	5’-TGGAG**G**-3’	KKH_SaCas9	NNNRRT	5’-TGGAG**T**-3’
		Fw	5’-**G**A-3’	Cas12c2	TN	5’-**T**A-3’
